# Barriers to rational antibiotic prescription in Iran: a descriptive qualitative study

**DOI:** 10.1186/s13756-022-01151-6

**Published:** 2022-08-29

**Authors:** Ramin Sami, Kobra Salehi, Raheleh Sadegh, Hamid Solgi, Vajihe Atashi

**Affiliations:** 1 Department of Internal Medicine, School of Medicine, Isfahan University of Medical Science, Isfahan, Iran; 2 Department of Midwifery and Reproductive Health, Nursing and Midwifery Care Research Center, School of Nursing and Midwifery, Isfahan University of Medical Science, Isfahan, Iran; 3 Department of Community and Preventive Medicine, School of Medicine, Isfahan University of Medical Science, Isfahan, Iran; 4 Isfahan Endocrine and Metabolism Research Center, Isfahan University of Medical Sciences, Isfahan, Iran; 5 Department of Adult Health Nursing, Nursing and Midwifery Care Research Center, School of Nursing and Midwifery, Isfahan University of Medical Science, Isfahan, Iran

**Keywords:** Antibiotic therapy, Rational antibiotic prescription, Barriers, Antimicrobial resistance, Infectious disease, Iran

## Abstract

**Introduction:**

Rational antibiotic prescription (RAP) refers to the purposeful and appropriate antibiotic prescription with correct dose and course to produce the most possible benefits and less possible side effects. Identification and management of the barriers to RAP can help promote RAP. The aim of the study was to explore the barriers to RAP in Iran.

**Methods:**

This descriptive qualitative study was conducted in 2021 on 46 physicians (including general physicians, specialists, and subspecialists), pharmacologists, microbiologists, and nurses. Participants were purposefully selected from five specialty and subspecialty hospitals in Isfahan, Iran, and the Treatment Administration of Isfahan University of Medical Sciences, Isfahan, Iran. Data were collected via semi-structured interviews and were analyzed via conventional content analysis.

**Results:**

The barriers to RAP in Iran came into sixteen subcategories and four main categories, namely physicians’ limited professional competence (with six subcategories), poor informational and functional resources (with four subcategories), ineffective supervision of RAP (with three subcategories), and inappropriate context for RAP (with three subcategories). The subcategories of these categories were physicians’ limited professional knowledge, physicians’ poor attitude towards RAP, physicians’ routine-based practice instead of evidence-based practice, physicians’ limited accountability, physicians’ fear over the legal consequences of not prescribing antibiotics, physicians’ financial motives, limited access to quality educational materials, poor in-service training for physicians, lack of culturally appropriate guidelines, inefficiency of the stewardship committee, limited supervision of physicians’ performance, ineffective managerial supervision, limited supervision of sampling for antimicrobial susceptibility testing, sociocultural factors contributing to irrational antibiotic prescription, poor adherence of insurance companies to their financial commitments, and financial incentives of pharmaceutical companies for physicians.

**Conclusion:**

The barriers to RAP are different and complex and include physician-related, resource-related, supervision-related, and contextual factors. Physicians with limited professional competence, limited access to resources, and limited supervision will have problems in RAP. Effective management of the barriers to RAP can promote RAP and minimize irrational antibiotic prescription and its consequences, chiefly antimicrobial resistance.

## Introduction

Antibiotics are the most commonly prescribed medications worldwide for mild to life-threatening infections [1]. In most countries, antibiotics constitute 30–50% of all prescribed medications [2]. A study reported that 54.9% of all prescriptions contained antibiotics [3].

Rational antibiotic prescription (RAP) is a key factor behand the effectiveness of antibiotic therapy [4]. By definition, RAP refers to the purposeful and appropriate antibiotic prescription with correct dose for patients with real need for antibiotic therapy to produce the most possible positive effects and less possible side effects [5]. RAP is among the key goals of the World Health Organization which considers RAP an effective strategy to reduce the morbidity and mortality of infections [6]. RAP is associated with different positive outcomes. For example, a study reported that the implementation of a RAP program consisted of biweekly educational seminars, auditing of physicians’ prescriptions for antibiotic, and provision of feedback to physicians reduced irrational antibiotic prescription (IRAP) from 43 to 20.6% and mortality rate from 10.4 to 8% [7, 8].

Despite the importance of RAP to patient survival and treatment outcomes, studies show a high IRAP prevalence of 30–60%. IRAP refers to the selection of wrong antibiotics with wrong doses or at wrong duration [9]. Studies show that most antibiotic prescriptions are inappropriate and the therapeutic value of 75% of antibiotics is questionable [10]. A study reported the poor status of antibiotic prescription and administration in Iran and noted that 58% of the prescribed antibiotics did not have rational indication [3]. Another study showed the over-prescription of antibiotics in Iran and the incongruence of antibiotic prescription with population conditions and infection prevalence. That study also reported that the rate of antibiotic use in Iran was almost equal to the total antibiotic use in Europe and sixteen times more than the global standards [11].

IRAP has many different negative outcomes. For instance, it reduces the quality of medication therapy, exposes patients to unnecessary medications, increases the risks of medication side effects, endangers patient safety, aggravates or prolongs ailment, imposes added financial burden, wastes resources, increases healthcare costs, and leads to antimicrobial resistance [12]. According to the Centers for Disease Control and Prevention (CDC), antimicrobial resistance in the United States increases healthcare-related costs by almost twenty billion dollars per year [13]. Infections resistant to antibiotics can in turn lead to ten million deaths by 2050 in the world [14].

Studies reported that RAP faces multiple barriers [15, 16]. A study showed public limited knowledge about RAP and antimicrobial resistance and physicians’ limited knowledge about antibiotic prescription guidelines as the barriers to RAP [17]. Another study also found that payments and incentives for physicians and number of patients referring to physicians affected RAP [18].

A prerequisite to the promotion of RAP is to explore its barriers in the immediate sociocultural context and healthcare conditions [19]. However, most studies in this area in Iran used quantitative designs to simply assess physicians’ knowledge and performance and hence, provided limited data, if any, about the barriers to RAP [11, 20]. The present study sought to narrow this gap. The aim of the study was to explore the barriers to RAP in Iran through a qualitative design. Qualitative studies provide more in-depth insight about phenomena [21].

## Methods

### Design

This descriptive qualitative study was conducted in 2021. Qualitative designs are used for the in-depth exploration and description of poorly known human-related phenomena such as concerns over events, responses to events, and facilitators or barriers to events in a certain sociocultural context. Descriptive qualitative designs enable researchers to explore phenomena which have direct effects on healthcare settings [22].

### Participants

Participants were 46 physicians, clinical pharmacologists, pharmacologist, microbiologists, and nurses purposefully selected with maximum variation respecting their age, gender, educational level, work experience, and organizational position. In purposive sampling, researchers select participants based on two main criteria, namely congruence between experience and research question and availability of the characteristics of a key informant [21]. Inclusion criteria were work experience more than two years and agreement for participation.

### Setting

Study setting consisted of five specialty and subspecialty hospitals in Isfahan, Iran, and the Treatment Administration of Isfahan University of Medical Sciences, Isfahan, Iran. This administration includes different committees such as the Stewardship Committee which is a subset of the Infection Control Committee. The Stewardship Committee was established in all hospitals of Iran in 2020 in order to control the use of costly antibiotics and prevent antimicrobial resistance in hospital settings. The members of the committee are hospital manager, an infectious disease specialist, an infection control nurse, a clinical pharmacologist, a clinical epidemiologist, and an information technologist. This committee is responsible for supervising antibiotic prescription in healthcare settings, developing guidelines for antibiotic prescription, implementing educational courses for physicians, analyzing antibiotic-related data, and comparing antibiotic-related indices. Each hospital has a Stewardship Committee which provides its reports about antibiotic prescription to the Stewardship Committee of the Treatment Administration of the affiliated university of medical sciences.

### Data collection

Data were collected through semi-structured interviews held in Persian from January to September 2021. Primarily, an interview guide was developed based on the study aim which included open-ended questions about the barriers to RAP. The content validity of the guide was confirmed by two nursing instructors experienced in qualitative research, a nurse with ten-year work experience in infection control, and a medical subspecialist who was familiar with qualitative research. Then, the guide was piloted and approved in two pilot interviews. Examples of the interview questions were “What is your general attitude towards antibiotic prescription?”, “What factors affect RAP?”, and “What are the barriers to RAP?”. Other interview questions were determined based on participants’ responses to the main interview questions. Besides, probing questions such as “What do you mean by this?”, “Can you provide more explanations about this?”, “Why”, and “How?” were used to further explore participants’ experiences. Participants were also asked to provide examples or explain the reasons of their responses in order to further clarify their experiences. At the beginning of each interview, the interviewee was informed that the aim of the interview was to explore his/her RAP-related opinions, perceptions, feelings, and experiences. Sampling and data collection were kept on up to data saturation, i.e., when no new data were obtained from the interviews. The place and the time of the interviews were determined according to participants’ preferences and the length of the interviews varied from twenty to sixty minutes according to their conditions. Interviews were audio-recorded using a digital voice recorder. The fifth author transcribed each interview word by word within at most 48 h after holding it.

### Data analysis

The fifth author analyzed the data concurrently with data collection using inductive conventional content analysis proposed by Graneheim and Lundman. Inductive content analysis is used when there are limited data about the intended phenomenon. In this approach, there is no predetermined categories; rather, categories are developed and labeled based on the raw data. The five steps of Graneheim and Lundman’s conventional content analysis are immediate transcription of each interview, perusal of the transcript to obtain a general understanding of the data, determination and coding of meaning units, categorization of the codes, and determination of the main categories [23]. For data analysis, the fifth author listened to the audio file of each interview and read its transcript several times in order to obtain a general understanding about its main ideas and immerse in the data. Then, she determined meaning units, i.e., words or expressions which related to the same central meaning [24] each identified meaning unit is labeled with a code, and coded them using participants’ or her own wording. Coding is actually the process of selective data reduction and simplification based on the study aim and facilitates concept determination [25]. Codes were grouped into subcategories based on their similarities and interrelationships and subcategories were similarly grouped into larger categories based on their interrelationships and similarities [23]. Categorization is the core of qualitative content analysis [24].

### Trustworthiness

Trustworthiness, which is equal to validity and reliability in quantitative studies, was ensured through the four criteria proposed by Guba and Lincoln, namely credibility, dependability, confirmability, and transferability [26]. Credibility was ensured through member checking, in which the transcript of the first five interviews and their codes were provided to their corresponding interviewees to check the congruence between the codes and their own experiences. All of them confirmed that our generated codes were congruent with their experiences. and dependability and confirmability were ensured through external peer checking by two experts in qualitative studies who assessed and confirmed the accuracy of data analysis. An audit trail was also created to ensure confirmability. The audit trail was created through documenting all steps of the study to provide others with the opportunity to track our study-related activities. Moreover, transferability was ensured through sampling with maximum variation and providing clear information about study participants and setting.

## Results

Participants were 36 physicians (including general physicians, specialists, and subspecialists), three clinical pharmacologists, one pharmacologist, three microbiologists, and three nurses (i.e., 46 in total) (Table 1). Four participants were twice interviewed because they announced in their first interview that they needed more time to think about the interview questions.

**Table 1:** Participants’ characteristics

No	Age (Years)	Gender	Occupation	Experience (Years)	No	Age (Years)	Gender	Occupation	Experience (Years)
1	48	Female	General physician	22	24	43	Female	Lung disease subspecialist	11
2	50	Female	Infectious disease specialist	26	25	52	Female	Clinical pharmacologist	21
3	57	Male	Infectious disease specialist	32	26	58	Female	Clinical pharmacologist	25
4	59	Female	Pharmacologist	30	27	60	Male	General surgery specialist	24
5	56	Male	Infectious disease specialist	24	28	42	Male	Orthopedic specialist	19
6	60	Male	Nephrology subspecialist	28	29	70	Male	Orthopedic specialist	35
7	55	Male	Nephrology subspecialist	23	30	45	Male	Lung disease subspecialist	18
8	63	Male	Anesthesiology subspecialist	30	31	38	Female	Lung disease subspecialist	12
9	68	Male	Internal medicine specialist	35	32	50	Male	General physician	25
10	67	Male	Internal medicine specialist	33	33	39	Female	General physician	14
11	51	Male	Lung disease subspecialist	14	34	45	Male	General physician	20
12	66	Male	Nephrology surgery specialist	31	35	39	Female	General physician	18
13	30	Female	General physician	3	36	28	Female	Infection control nurse	6
14	32	Female	Internal medicine specialist	4	37	37	Female	Infection control nurse	12
15	38	Female	Infectious disease specialist	8	38	26	Female	General physician	2
16	39	Female	Internal medicine specialist	9	39	37	Female	Microbiologist	13
17	45	Female	Internal medicine specialist	14	40	45	Female	Microbiologist	20
18	37	Male	Internal medicine specialist	7	41	32	Male	Microbiologist	5
19	41	Male	Clinical pharmacologist	15	42	66	Male	Thoracic surgery subspecialist	27
20	43	Male	Infectious disease specialist	13	43	64	Male	General surgery specialist	22
21	45	Female	Hematology and oncology subspecialist	13	44	58	Male	General physician	33
22	50	Male	Cardiac surgery subspecialist	16	45	42	Male	General physician	17
23	52	Male	Gastroenterology and hepatology subspecialist	18	46	32	Female	Infection control nurse	10

The barriers to RAP in Iran were grouped into four main categories, namely physicians’ limited professional competence, poor informational and functional resources, ineffective supervision of RAP, and inappropriate context for RAP (Table 2).

**Table 2:** The subcategories and categories of the barriers to rational antibiotic prescription in Iran

Subcategories	Categories
Physicians’ limited professional knowledge	Physicians’ limited professional competence
Physicians’ poor attitude towards RAP	
Physicians’ routine-based practice instead of evidence-based practice	
Physicians’ limited accountability	
Physicians’ fear over the legal consequences of not prescribing antibiotics	
Physicians’ financial motives	
Limited access to quality educational materials	Poor informational and functional resources
Poor in-service training for physicians	
Lack of culturally appropriate guidelines	
Inefficiency of the stewardship committee	
Limited supervision of physicians’ performance	Ineffective supervision of RAP
Ineffective managerial supervision	
Limited supervision of sampling for antimicrobial susceptibility testing	
Sociocultural factors contributing to IRAP	Inappropriate context for RAP
Poor adherence of insurance companies to their financial commitments	
Financial incentives of pharmaceutical companies for physicians	

### Physicians’ limited professional competence

Participants highlighted the significant effects of physicians’ professional knowledge, skills, and attitude on RAP. This category had six subcategories, namely physicians’ limited professional knowledge, physicians’ poor attitude towards RAP, physicians’ routine-based practice instead of evidence-based practice, physicians’ limited accountability, physicians’ fear over the legal consequences of not prescribing antibiotics, and physicians’ financial motives.

#### Physicians’ limited professional knowledge

Participants noted that tasks cannot correctly be understood and performed without adequate knowledge about them and thereby, considered knowledge as a prerequisite to RAP. Nonetheless, their experiences showed that physicians had limited knowledge about RAP.

Residents and interns cannot provide good answers when I ask them about sepsis and why they prescribe vancomycin, meropenem, or ceftazidime for it. They usually start broad-spectrum antibiotics instead of simple antibiotics (P. 40).

#### Physicians’ poor attitude towards RAP

Participants reported that attitude and beliefs have significant effects on performance and noted that some physicians do not prioritize RAP due to their poor attitude towards it.

The problem is that our physicians have no sensitivity towards the risks of IRAP and do not care the consequences of IRAP for patients. I have even seen that some colleagues freely prescribe injectable antibiotics for very simple infections (P. 3)

#### Physicians’ routine-based practice instead of evidence-based practice

Some participants’ experiences showed that antibiotics were prescribed based on routines rather than patients’ needs, the results of laboratory tests, or the opinions of infectious disease specialists.

Unfortunately, we are affected by routines and pay no attention to the results of patients’ laboratory tests. It is very likely to prescribe ceftriaxone for all patients of a single ward, even a patient with asthma (P. 24).

#### Physicians’ limited accountability

Most participants expressed that some physicians do not adhere to the existing guidelines for antibiotic prescription, do not document the reasons for antibiotic therapy in patients’ medical records, and do not allocate adequate time to provide patients with information about the reasons for prescribing or not prescribing antibiotics.

A recent organizational rule requires physicians to document the reasons for starting antibiotic therapy or changing antibiotic regimen in patients’ medical records. Nonetheless, I haven’t so far seen physicians’ adherence to this rule and they even provide no good answer when you ask them about such non-adherence (P. 37).

#### Physicians’ fear over the legal consequences of not prescribing antibiotics

Some participants noted that physicians freely prescribe broad-spectrum antibiotics from the very beginning of treatment due to the pressures of the mortality committees of hospitals and their fear over legal prosecutions.

Now, the mortality committees of hospitals impose added pressures on physicians and hence, physicians freely use broad-spectrum antibiotics even in spite of no definite diagnosis in order not to experience legal problems (P. 10).

#### Physicians’ financial motives

Some participants reported that physicians freely prescribe antibiotics in order to satisfy their patients, attract more patients, and have more income.

A main reason for IRAP is physicians’ financial motives. Practically, physicians’ income in both public and private centers directly depends on the number of their visited patients and hence, they may prescribe antibiotics just to satisfy their patients (P. 2).

### Poor informational and functional resources

Participating physicians reported that they needed adequate knowledge for RAP and highlighted that such knowledge can be acquired through educational programs, clear guidelines, and acts of stewardship committees. However, participants’ experiences revealed that physicians had problems in accessing informational resources such as quality educational materials, quality in-service training, and culturally appropriate guidelines. They also noted that stewardship committees had poor performance in providing quality informational resources to physicians.

#### Limited access to quality educational materials

Participants’ experiences showed that physicians had limited access to up-to-date evidence and informational resources for RAP and there was no serious planning to improve their access to such resources.

I can better decide on antibiotic prescription if I have adequate knowledge and information. However, my limited access to up-to-date resources and articles limits my prescriptions to my experience and routines (P. 17).

#### Poor in-service training for physicians

According to the participants, in-service RAP-related training for physicians has many positive outcomes such as improvement of RAP-related knowledge and quality of medication therapy. However, participating physicians reported that they had not received effective in-service RAP-related training and their RAP-related knowledge was limited to the educations received during their studentship.

Physicians start antibiotic therapy for fever but do not know the antibacterial spectrum of tazocin due to our low quality education. In my opinion, all physicians, from general physicians to subspecialists, need in-service training (P. 41).

#### Lack of culturally appropriate guidelines

Participants expressed that updated culturally appropriate guidelines with clear explanations are needed for RAP.

At first, guidelines should be developed. Of course, I think that foreign guidelines are not useful for us because the type of microbes varies according to each setting. Moreover, guidelines should annually be revised (P. 11).

#### Inefficiency of the stewardship committee

Participants highlighted that the Stewardship Committee was one of the most important and most essential hospital committees for RAP promotion. However, their experiences showed that the sessions of the committee were not regularly held, its acts were not followed, and key individuals such as infectious disease specialists, clinical pharmacologist, and microbiologists, did not serve on it.

Hospital managers don’t actively attend the committee. Moreover, an infectious disease specialist should attend the committee. I want to say that this committee does not effectively address RAP-related problems and issues and its existence is just for bureaucratic purposes (P. 46).

### Ineffective supervision of RAP

According to the participants, supervision is a significant factor in performance improvement which helps ensure the accurate performance of tasks and activities. Some participants reported managerial supervision as one of their motives for RAP. This category had three subcategories, namely limited supervision of physicians’ performance, ineffective managerial supervision, and limited supervision of sampling for antimicrobial susceptibility testing.

#### Limited supervision of physicians’ performance

Participants’ experiences showed limited supervision and feedback respecting physicians’ performance in antibiotic prescription. Moreover, they highlighted that there was no reaction to physicians’ IRAP.

The stewardship committee has poor performance, has no regular sessions, superficially assesses the free use of broad-spectrum antibiotics, and never asks physicians about their use of broad-spectrum antibiotics (P. 26).

#### Ineffective managerial supervision

Some participants noted that their multiple responsibilities reduced their ability to effectively perform their supervisory responsibilities.

As the hospital manager and a member of the Stewardship Committee, I’m so involved in performing hospital affairs that cannot effectively perform my principal supervisory task in the committee (P. 8).

#### Limited supervision of sampling for antimicrobial susceptibility testing

Participants’ experiences also showed that the quality of sampling and bacterial culture can affect RAP. They highlighted that nurses had poor adherence to the principles of bacterial culture and there was limited supervision on their sampling practice.

I can’t trust the results of antimicrobial susceptibility testing and can’t use them for antibiotic therapy because I have seen that there is no supervision of the sampling process and there is limited adherence even to the principles of sterility during sampling. Therefore, I prescribe antibiotics based on my own clinical judgment (P. 9).

### Inappropriate context for RAP

Contextual factors can also act as barriers to RAP. The three subcategories of this category were sociocultural factors contributing to IRAP, poor adherence of insurance companies to their financial commitments, and financial incentives of pharmaceutical companies for physicians.

#### Sociocultural factors contributing to IRAP

Participants’ experiences indicated public perception and image of antibiotics as a major factor contributing to IRAP. Participants highlighted that during medical visits, patients usually expect physicians to prescribe antibiotics for them. Thus, they will refer to another physician or start over-the-counter use of antibiotics if their physicians do not prescribe antibiotics.

Public health literacy and culture are very important. Some people referred to me from far distances and insisted on antibiotic prescription. I don’t like to say that I was obedient to them, did not resist against their insistence on antibiotic prescription, and prescribed antibiotics for them (P. 33).

#### Poor adherence of insurance companies to their financial commitments

Some participants reported delayed payments of insurance companies as a major barrier to RAP.

Insurance companies have long delays, even for six months, in paying doctors for their prescriptions. This moves physicians towards the prescription of more medications (P. 3).

#### Financial incentives of pharmaceutical companies for physicians

Participants reported that pharmaceutical companies provide financial incentives to physicians for the prescription of their manufactured antibiotics and highlighted that such incentives move physicians towards IRAP.

Sometimes, pharmaceutical companies offer foreign tours to physicians in order to motivate them to prescribe their medications (P. 26).

## Discussion

This study explored the barriers to RAP in Iran. Findings revealed that the four main barriers to RAP in Iran were physicians’ limited professional competence, poor informational and functional resources, ineffective supervision of RAP, and inappropriate context for RAP.

Physicians’ limited professional competence was one of the major barriers to RAP in Iran. Physicians’ limited professional knowledge was one of the subcategories of this category. In line with this finding, a study in China reported that physicians had moderate knowledge about antibiotic prescription due to factors such as non-participation in in-service training courses respecting antibiotic prescription in the past one year [27]. Another study in Congo found that physicians had limited knowledge about antibiotic prescription and antimicrobial resistance [28]. Limited attention to the development and use of quality educational policies for medical students and physicians both during university education and after graduation can lead to their limited knowledge about medications. Physicians’ poor attitude towards RAP was another subcategory of the physicians’ limited professional competence main category. This finding highlights that adequate knowledge about RAP may not guarantee RAP in practice. Thus, interventions for modifying attitudes and beliefs are needed to promote RAP. A previous study in Iran found that while physicians reported antimicrobial resistance as a global problem, more than half of them prescribed antibiotics in their daily practice probably due to their limited sensitivity to the risks of IRAP and poor understanding about the importance of RAP [29]. Our findings also showed that some physicians did not value the results of antimicrobial susceptibility testing, did not consult infectious disease specialists, pharmacologists, and microbiologists for antibiotic prescription, and prescribed antibiotics based on the existing routines. Similarly, a previous study in Iran reported that 5.6% of physicians never consulted their colleagues and 48% of them occasionally consulted their colleagues for antibiotic therapy [29]. Effective communication between pharmacologists and physicians can reduce the barriers to RAP [30, 31]. Clinical pharmacologists can provide physicians with accurate information about medications and thereby, help them select the best antibiotics for patients, increase the effectiveness of antibiotic therapy, and promote RAP [32]. Given the wide spread of infectious diseases, the need for antibiotic therapy, and high prevalence of inappropriate use of medications, physicians need to more closely consult with infectious disease specialists and pharmacologists to select and prescribe the most appropriate antibiotics. Physicians’ limited accountability was another physician-related barrier to RAP in the present study. In agreement with this finding, a previous study found physicians’ professional commitment, accountability, and adherence to professional ethics as factors contributing to RAP [33]. Another study also showed effective physician–patient communication as a key factor in RAP [34]. Moreover, our findings indicated physicians’ financial motives as a physician-related barrier to RAP. In other words, physicians prescribed medications to attract, satisfy, and keep their clients and prevent potential financial losses. Similarly, a previous study reported commercial approach to treatment as a factor with significant contribution to RAP. Such approach has created a marketing atmosphere in medicine with unhealthy competition among healthcare providers for more financial benefits which endangers patients’ benefits [18]. Therefore, more intensive supervision of physicians’ practice, particularly antibiotic therapy, is necessary to promote RAP and minimize IRAP and its consequences.

Study findings also revealed poor informational and functional resources as the second main barrier to RAP in Iran. A study in Iran reported that 47% of physicians had not participated in educational programs on antibiotic prescription and 95% of them agreed with the implementation of such programs [29]. Some previous studies showed in-service training courses, online education, provision of clinical guidelines, sharing experiences, and face-to-face education as effective instructional strategies with significant positive effects on the quality of physicians’ antibiotic prescriptions [35, 36]. Lack of culturally appropriate guidelines for RAP was a resource-related barrier to RAP in the present study. A study reported that evidence-based guidelines are essential for RAP promotion [37]. Moreover, we found inefficiencies in the performance of the Stewardship Committee, such as non-inclusion of key experts, no constructive feedback to physicians, and no supervision of the use of guidelines and acts, as a significant barrier to RAP. This is in line with the findings of a previous study which reported inefficiencies in the performance of the Stewardship Committee, such as limited participation of physicians, lack of quality diagnostic equipment, lack of official mechanisms for data collection, and lack of effective strategies for interdisciplinary collaboration, as major barriers to the effective management of antimicrobial resistance [38].

The third main barrier to RAP was ineffective supervision of RAP. Findings showed that physicians’ RAP practice was symbolically and inadequately supervised mostly for bureaucratic purposes and its results had no significant effects on physicians’ career advancement. Supervision and feedback are key factors in motivating physicians for behavior modification respecting RAP [39]. A study reported that regularly auditing and supervising physicians’ antibiotic therapy practice and providing them with constructive feedback had significant role in reducing antibiotic prescription [5]. Our findings also revealed that some managers had multiple roles and hence, had limited time to supervise physicians’ antibiotic therapy. Similarly, a previous study in Iran reported that managers’ heavy workload due to their role multiplicity limited their time for effective supervision [40].

Inappropriate context for RAP was the fourth main category of the barriers to RAP. Study findings showed that some sociocultural factors such as public limited knowledge, habitual use of antibiotics, and patients’ insistence on antibiotic prescription contributed to IRAP. Medication prescription is a complex process affected by sociocultural factors [29] such as patients’ educational level, expectations, and previous experiences of medication use [41]. The physicians’ paternalistic approach in healthcare systems in Iran, their ineffective communication with pharmacologists and other healthcare providers, and their tendency towards satisfying their patients are among the most important factors contributing to IRAP [42]. Moreover, our findings showed the inefficiency of the policies and strategies of insurance companies for paying to physicians as a major factor contributing to IRAP. The debt of insurance companies to physicians and pharmacies is a major challenge in the healthcare system of Iran which can lead to public dissatisfaction with healthcare services and irrational use of medications. A study in Iran found that long-term debts of insurance companies make physicians use illegal strategies to compensate their financial losses [18]. Moreover, we found that the financial incentives of pharmaceutical companies for physicians contributed to IRAP. Pharmaceutical companies introduce their products to the market through physicians and pharmacologists, which can lead to IRAP by physicians. These companies also attempt to influence all their customers, including physicians and pharmacologists, through offering free products, holding congresses and seminars, and placing advertisements in medical journals and media [43]. Therefore, governmental organizations and authorities need to closely supervise pharmaceutical companies and their interactions with physicians to reduce the devastating effects of their inappropriate interactions on medication prescription and use.

Based on the findings of the present study, strategies such as development of culturally appropriate guidelines for antibiotic prescription, promotion of medical professionalism, development of professional guidelines for ethical practice, closer governmental supervision of the practice of physicians, pharmacies, and pharmaceutical companies, development of the financial abilities of insurance companies, and improvement of public financial status are recommended to improve the quality of antibiotic prescription and medical care [44]. In low and middle income countries like Iran, which suffer from resource shortage, public education about RAP and the complications of over-the-counter use of antibiotics may be needed to overcome the challenges of RAP. Moreover, public education through media and medical sciences universities is needed to correct the existing misconceptions about antibiotic use. In-service training for physicians about antimicrobial resistance the risks of IRAP are also recommended to promote RAP in these countries [45].

## Strengths

This study was among the first qualitative studies into the barriers to RAP in Iran. Its participants were different individuals involved in antibiotic therapy, namely physicians, pharmacologists, clinical pharmacologists, microbiologists, and nurses.

## Limitations

Five participants did not consent to audio-record their interviews and hence, their interviews were transcribed, which led to the loss of some data.

## Conclusion

This study suggests the multiplicity of the barriers to RAP in Iran which include physicians’ limited professional competence, poor informational and functional resources, ineffective supervision of RAP, and inappropriate context for RAP. The findings of the present study provide a new understanding of the barriers to RAP for governmental and healthcare authorities and can be used to develop effective interventions to promote RAP and reduce the complications of IRAP, mainly antimicrobial resistance. These interventions may include clear guidelines for antibiotic therapy, purposeful educational interventions, coherent and close supervision of antibiotic therapy, in-service RAP-related training courses for physicians, and patient and public education about appropriate use of antibiotics.

## Data Availability

The datasets analyzed during the current study available from the corresponding author on reasonable request.

## Abbreviations

RAP: Rational antibiotic prescription
IRAP: Irrational antibiotic prescription
CDC: Centers for disease control and prevention
